# Ubiquitin-like protein-mediated sulfur trafficking facilitates hyperthermophile dispersal in sulfur-limited environments

**DOI:** 10.1128/mbio.01072-25

**Published:** 2025-07-01

**Authors:** Peng Zhou, Xue-Qing He, Qi-Liang Lai, Yue-Hong Wu

**Affiliations:** 1 State Key Laboratory of Submarine Geoscience, Second Institute of Oceanography, Ministry of Natural Resources 118476, Hangzhou, People's Republic of China; 2 Key Laboratory of Marine Genetic Resources, Third Institute of Oceanography, Ministry of Natural Resources, Xiamen, People's Republic of China; NYU Langone Health, New York, New York, USA

**Keywords:** hyperthermophile, hydrothermal vent, dispersal, cysteine desulfurase, ubiquitin-like proteins

## Abstract

While hyperthermophilic archaea thriving in hydrothermal vent ecosystems have been extensively studied for their remarkable adaptations to geochemical extremes, the molecular underpinnings of their dispersal strategies remain enigmatic. Central to this challenge lies their capacity to survive in environments with limited elemental sulfur (S^0^). The recent study by Hidese et al. (mBio 15:e00534-24, 2024, https://doi.org/10.1128/mbio.00534-24) provides critical mechanistic insights by elucidating the functional roles of three ubiquitin-like proteins (Ubls) as sulfur carriers in *Thermococcus kodakarensis*. These Ubls facilitate metabolic flexibility in sulfur utilization, and the Ubl-involved sulfur relay system represents an elegant adaptive solution for persistence in S^0^-limited niches. By enabling efficient sulfur mobilization from cysteine stores, the organism achieves metabolic homeostasis under S^0^ deprivation, such as biosynthesis of essential biomolecules. These findings not only reveal a previously unrecognized adaptive paradigm in sulfur utilization among hyperthermophilic archaea but also underscore the importance of metabolic plasticity for microbial dispersal and evolution.

## COMMENTARY

Hyperthermophilic microorganisms thrive in dynamic hydrothermal vent ecosystems globally, serving as model systems for investigating ecological adaptability and habitat colonization due to their extreme environmental niche characterized by elevated temperatures and elemental sulfur (S^0^) abundance (1, 2). While dispersal mechanisms are critical for hyperthermophile transmission, particularly before colonization of nascent hydrothermal vent systems, fundamental knowledge gaps remain regarding the metabolic architectures that enable these organisms to traverse environmental gradients beyond their primary vent habitats.

Although hyperthermophiles exhibit remarkable cryptobiosis capabilities, surviving years in cold environments (3) and at low temperatures may slow down their metabolism, resulting in their ability to survive during dispersion (1); marine ecosystems exhibit pronounced spatiotemporal heterogeneity. Thermal regimes, in particular, frequently exceed psychrophilic thresholds, especially in shallow-water habitats, creating significant physiological challenges during dispersal. Elucidating the molecular underpinnings of this metabolic plasticity is therefore pivotal to deciphering the strategies that facilitate hyperthermophile range expansion and ecosystem colonization in fluctuating marine environments.

A paramount adaptive challenge resides in the metabolic reprogramming required for sulfur source transitions, exemplified by the shift from S^0^-repleted hydrothermal vent environments to S^0^-depleted marine waters. The adaptation to sulfur source transition is crucial because sulfur participates in biomolecules that are involved in numerous essential cellular processes, including DNA repair, respiration, intermediary metabolism, gene regulation, and redox sensing (4). Despite modern seawater contains ~28 mM sulfate (5) as a potential sulfur reservoir, its metabolic incorporation via energetically costly pathways—such as the ADP-demanding sulfate adenylyltransferase-catalyzed reaction (6)—appears suboptimal for sustaining dispersal-related metabolic demands. While assimilatory sulfate reduction has been increasingly documented in marine microorganisms (e.g., *Methanothermococcus thermolithotrophicus*) (7), the potential for *T. kodakarensis* to utilize sulfate as a sulfur source under specific conditions warrants further investigation. However, a critical unresolved question persists: do hyperthermophilic archaea employ energy-sparing mechanisms to directly mobilize intracellular sulfur reserves for the biosynthesis of sulfur-containing biomolecules?

A recent study by Hidese et al. (8) has significantly advanced our understanding of both functional diversification and physiological relevance of three ubiquitin-like proteins (Ubls) in sulfur metabolic pathways within the hyperthermophilic archaeon *Thermococcus kodakarensis*. Through systematic mutagenesis and comparative phenotyping, the research elucidates distinct functional roles for the three Ubls (TK1065, TK1093, and TK2118) in orchestrating biosynthesis of sulfur-containing biomolecules. Specifically, TK1093 plays an important role in the efficient thiolation of tRNAs, which contributes to cellular thermotolerance, whereas TK2118 is involved in the biosynthesis of the tungsten cofactor within glyceraldehyde 3-phosphate:ferredoxin oxidoreductase, which is required for glycolytic growth. Intriguingly, Hidese et al. also demonstrated that the Ubl mutants restored defective synthesis of these sulfur-containing molecules with the presence of elemental sulfur in the culture medium, indicating that *T. kodakarensis* can use S^0^ as an alternative sulfur source without Ubls (8). These findings provide novel insights into metabolic plasticity mechanisms that facilitate environmental adaptation and dissemination in marine ecosystems. Notably, it has been previously verified by Hidese et al. that cysteine desulfurase (CD) transfers the sulfur atom of L-cysteine to sulfur-carrier proteins and plays an important role in the environmental adaptation of *T. kodakarensis* (9). Collectively, these findings underscore that the CD- and Ubl-mediated redundant sulfur trafficking from L-cysteine enables *T. kodakarensis* to overcome S^0^ limitation, facilitating survival across sulfur-variable ecosystems.

Furthermore, phylogenetic analyses reveal a broad distribution of CD orthologs across diverse archaeal lineages (9), and the three Ubl genes exhibit high evolutionary conservation in archaea (8, 10). Building on these observations, we proposed that during microbial dispersal events, the microorganisms harboring CD may convert L-cysteine to L-alanine and sulfane sulfur through CD-catalyzed reactions, generating protein-bound cysteine persulfide intermediates. These activated sulfur species could subsequently be channeled into diverse biosynthetic pathways via enzymatic catalysts, such as Ubls, enabling the production of essential sulfur-containing biomolecules like molybdopterin, tungsten cofactor, and tRNA thiouridines.

Beyond archaea, Ubl orthologs exhibit a broad taxonomic distribution, encompassing diverse eukaryotes and bacteria (8). These proteins mediate sulfur trafficking for the biosynthesis of sulfur-containing biomolecules and form conjugates with specific protein targets to regulate their functions (8). Notably, the deletion mutants generated by Hidese et al. (8) now establish a sophisticated genetic framework for systematic functional dissection of the extensive Ubl orthologs. Moreover, S^0^-limited environments are ubiquitous on the Earth, hosting numerous organisms. The sulfur-trafficking pathways mediated by Ubls in *T. kodakarensis* enable the biosynthesis of sulfur-containing biomolecules under low-S^0^ conditions. Consequently, investigating the evolutionary origins of genes responsible for encoding Ubls, along with their acquisition processes and functional redundancy, has the potential to shed light on the underlying mechanisms governing the adaptive evolution of life. Furthermore, it could provide insights into the expansion of habitable zones, ranging from S^0^-rich environments like hydrothermal vents to S^0^-depleted settings such as oxic seawaters.

In conclusion, the seminal works by Hidese et al. offer profound insights into the intricate metabolic machinery of microorganisms and their evolutionary adaptive strategies for environmental dissemination. As ongoing functional characterization continues to decode the metabolic roles of additional genetic elements, the molecular underpinnings of microbial fitness across heterogeneous ecosystems will be progressively unveiled. These advances not only refine our mechanistic understanding of microbial physiology but also open transformative frontiers for deciphering the principles governing microbial dispersal in diverse geochemical settings.
